# The Impact of Glucose-Lowering Strategy on the Risk of Increasing Frailty Severity among 49,519 Patients with Diabetes Mellitus: A Longitudinal Cohort Study

**DOI:** 10.14336/AD.2023.0225

**Published:** 2023-10-01

**Authors:** Chun-Yi Chi, Jui Wang, Szu-Ying Lee, Chia-Ter Chao, Kuan-Yu Hung, Kuo-Liong Chien

**Affiliations:** ^1^Nephrology division, Department of Internal Medicine, National Taiwan University Hospital Yunlin branch, Yunlin County, Taiwan.; ^2^Institute of Epidemiology and Preventive Medicine, College of Public Health, National Taiwan University, Taipei, Taiwan.; ^3^Health Management Center, National Taiwan University Hospital, Taipei, Taiwan.; ^4^Nephrology division, Department of Internal Medicine, National Taiwan University Hospital, Taipei, Taiwan.; ^5^Nephrology division, Department of Internal Medicine, National Taiwan University College of Medicine, Taipei, Taiwan.; ^6^Graduate Institute of Toxicology, National Taiwan University College of Medicine, Taipei, Taiwan

**Keywords:** diabetes mellitus, frail phenotype, frailty, glucose-lowering drugs

## Abstract

Patients with diabetes mellitus (DM) have a higher risk of incident and aggravating frailty over time. Frailty-initiating risk factors have been identified, but modulators of frail severity over time remain poorly defined. We aimed to explore the influences of glucose-lowering drug (GLD) strategy on DM patients’ risk of increasing frail severity. We retrospectively identified type 2 DM patients between 2008 and 2016, dividing them into “no GLD”, oral GLD (oGLD) monotherapy, oGLD combination, and those receiving insulin without or with oGLD at baseline. Increasing frail severity, defined as ≥1 FRAIL component increase, was the outcome of interest. Cox proportional hazard regression was utilized to analyze the risk of increasing frail severity associated with GLD strategy, accounting for demographic, physical data, comorbidities, medication, and laboratory panel. After screening 82,208 patients with DM, 49,519 (no GLD, 42.7%; monotherapy, 24.0%; combination, 28.5%; and insulin user, 4.8%) were enrolled for analysis. After 4 years, 12,295 (24.8%) had increasing frail severity. After multivariate adjustment, oGLD combination group exhibited a significantly lower risk of increasing frail severity (hazard ratio (HR) 0.90, 95% confidence interval (CI) 0.86 - 0.94), while the risk of insulin users increased (HR 1.11, 95% CI 1.02 - 1.21) than no GLD group. Users receiving more oGLD exhibited a trend of less risk reduction relative to others. In conclusion, we discovered that the strategy of oral glucose lowering drugs combination might reduce the risk of frail severity increase. Accordingly, medication reconciliation in frail diabetic older adults should take into account their GLD regimens.

## INTRODUCTION

Frailty, a geriatric phenotype that describes the accumulation of subclinical deficits in multiple dimensions, characterizes vulnerable individuals at risk of premature mortality. Since the conceptualization and operationalization of frailty nearly 2 decades ago [1], its plausible etiologies, biological correlates and clinical impact have gradually been unraveled [2]. Cellular aging, or senescence, and the associated subcellular alterations involving mitochondria, methylation profile, and telomere length are preceding microscopic events before frank frailty development [3]. The theory of the hallmarks of aging [4] attempts to provide a summary of aging-related changes, and the resultant dysregulation of bodily homeostasis and homeodynamics lead to the subsequent occurrence of prefrailty and later frailty. Frailty has now evolved to encompass different subtypes, such as cognitive frailty, oral frailty, social frailty, etc., each with its unique pathophysiology and health effects. A systematic review reported that having frailty increased the risk of mortality and disability by approximately 2- and 6-fold among older adults [5]. Other meta-analyses showed that frail older individuals were further at risk of prolonged hospital stay and functional decline in the long run [6].

Apart from older adults, patients with diabetes mellitus (DM) are similarly prone to developing frailty. Dysglycemia, including hypoglycemic episodes [7] or high glycated hemoglobin levels [8], was a significant risk factor for frailty occurrence, while DM at an earlier period also elevated the risk of developing frailty years later [9]. Patients with DM tend to have sarcopenia, compromised muscular strength/muscle quality while reduced muscle mass, accompanied by multimorbidity involving the kidney, heart, peripheral and cerebral vessels. Vascular structure and function are frequently jeopardized during DM, in combination with insulin resistance and chronic inflammation, leading to physical impairment among affected individuals. Indeed, epidemiological surveys yielded that DM raised the probability of developing frailty, augmented further by hypertension or other diabetic complications [10].

Several risk factors modulate the risk of frailty in patients with DM. Wang *et al*. revealed that diabetic complications such as chronic kidney disease (CKD) and vascular morbidities affected the risk of incident frailty at different timings following DM onset [11]. Oral function integrity and its associated factors, including chewing capacity, choking probability, and tooth brushing habits, also play a role in determining the risk of frailty in patients with DM [12]. Existing studies mainly address factors driving the initiation of frailty in this population; however, few studies focused on modifiable risk factors regarding the dynamic changes in frailty over time. This is particularly of concern, as the frailty prevalence in this population increases successively over time, precluding the administration of early preventive strategies, and the temporal change in frail severity substantially predisposes patients to mortality [13]. It is therefore important that we identify potentially ameliorable factors for retarding frailty progression in patients with DM. Glucose-lowering drug (GLD) regimen adjustment may be one of the candidates. A prior study did disclose that different glucose-lowering strategies affected the risk of cardiovascular events in this population [14]. We hypothesized that glucose-lowering strategy might influence the probability of frail severity progression among patients with DM. In this study, we harnessed a prospectively assembled institution-based cohort to examine such issues.

## MATERIALS AND METHODS

### Ethical statement

The protocol of the current study, as part of a comprehensive project focusing on the epidemiology of DM, has been approved by the institutional review board of National Taiwan University Hospital (NTUH NO. 201708098RIND). This study was carried out with adherence to the Declaration of Helsinki. Informed consent was waived by the institutional review board due to our study’s retrospective nature.

### Enrollment of study participants

Participants of this study were retrospectively identified from NTUH integrated medical database (NTUH-iMD) between 2008 and 2016. Inclusion criteria were participants older than 40 years with at least 1 time of physician-diagnosed type 2 DM at out-patient, in-patient, or the emergency department, with a glycated hemoglobin ≥6.5% or a fasting glucose ≥126 mg/dL. Exclusion criteria consisted of those with missing laboratory data, with type 1 DM, without subsequent follow-up, or those with the outcome of interest, which was specified below, at baseline.

Participants were further subdivided into those without receiving any GLD during the entire follow-up period (“no GLD” group), those who received only one type of oral GLD (oGLD) (“oGLD monotherapy” group) or a combination of at least 2 oGLD at baseline (“oGLD combination” group), and those receiving insulin without or with oGLD (“insulin user” group). Medication use was defined according to the filling of prescriptions ≥28 days within 1 year after the date of DM diagnosis. We recorded their clinical features, including demographic factors (age and sex), body mass index (BMI), comorbidities, concurrent medications (anti-hypertensives, lipid-lowering agents, anti-platelets, and anticoagulants), and laboratory profiles (fasting glucose, glycated hemoglobin, total cholesterol and triglyceride, and serum creatinine). We calculated their estimated glomerular filtration rate (eGFR) based on the 4-variable Modification of Diet in Renal Disease (MDRD) formula. Participants were longitudinally followed up from the index date, which was 1 year after DM diagnosis, until the development of frailty or the end of follow-up, which was Dec 31^st^, 2017.

### Outcome definition

In this study, the aggravation of frail severity was the outcome of interest. We assessed frailty based on the modified FRAIL scale, comprised of 5 components (fatigue, resistance, ambulation, illness, and loss of weight) [15]. FRAIL scale was coined since 2012 as an easy-to-administer instrument for identifying frail adults, and its results exhibited excellent correlation with those using the Fried’s criteria [16] and other instruments [17]. Individuals with FRAIL-detected frailty (≥3 positive components) had an increased risk of mortality, disability, fall, institutionalization, and other geriatric syndrome development compared to robust ones [18-20]. FRAIL scale has been validated for its applicability in younger populations, including those with morbidities such as CKD or DM [21]. We further operationalized the FRAIL scale based on physical and diagnostic combinations outlined previously [22]. Prior studies also showed that increasing FRAIL component was predictive of rising risk of mortality, healthcare utilization, and multiple organ morbidities [23,24], and thus this phenomenon may correlate with rising frail severity.

### Statistical analysis and analytic plan

For descriptions of continuous and categorical variables, we used means ± standard deviations or numbers with percentages, respectively. We compared continuous and categorical variables using the Student’s *t*-test and the chi-square test, respectively. We first compared demographic profile, physical data, comorbidities, medication regimens, and laboratory panels between the no GLD, oGLD monotherapy, oGLD combination, and the insulin user groups. Kaplan-Meier technique was used to construct event incidence curves of different treatment groups. This was followed by univariate and multivariate Cox proportional hazard regression analyses, with rising frail severity, or the increase of ≥1 positive components as the dependent variable. We analyzed the risk of rising frail severity associated with glucose lowering drug strategy, incorporating all clinical variables examined above, using hazard ratio (HR) and 95% confidence interval (CI). Several sensitivity analyses were conducted, including one that focused on those without any FRAIL component at baseline, another focusing on the type of GLDs individually, and the other focusing on eGFR influences. During all analyses, a two-tailed p value <0.05 was considered statistically significant.

**Figure 1.**
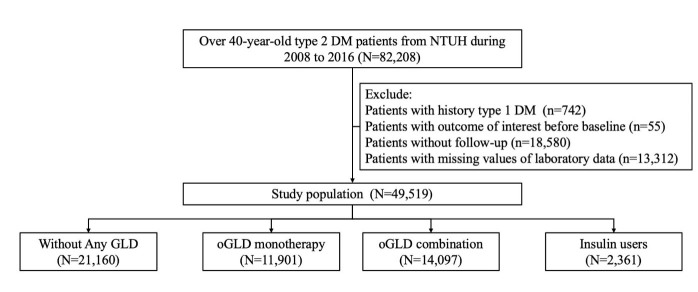
Flow chart of study participants selection. DM, diabetes mellitus; GLD, glucose lowering drug; NTUH, National Taiwan University Hospital; oGLD, oral glucose lowering drug.

## RESULTS

A total of 82,208 patients with DM were initially screened for eligibility, and after applying the exclusion criteria, 49,519 patients with type 2 DM were enrolled for subsequent analysis (Fig. 1). They were divided into the no GLD group (n=21,160; 42.7%), oGLD monotherapy group (n=11,901; 24.0%), oGLD combination group (n=14,097; 28.5%), and the insulin user group (n=2,361; 4.8%). Patients with DM and receiving oGLD monotherapy and insulin users had significantly higher age (*p* <0.001), while those who were insulin users and those without receiving any GLD were more likely to be female (*p* <0.001) (Table 1). The oGLD monotherapy group had the highest prevalence of hypertension, hyperlipidemia, atrial fibrillation, and cerebrovascular accident (all *p* <0.001) than the other groups. On the other hand, insulin user group had the highest prevalence of cancer, prior myocardial infarction, heart failure, peripheral vascular disease, and CKD (all *p* <0.001) than the other groups. Insulin users more commonly received anti-hypertensives and lipid-lowering agents, while oGLD monotherapy and oGLD combination groups most commonly received anticoagulants and anti-platelet agents, respectively (*p* <0.001) (Table 1). Regarding laboratory panel, insulin users had the highest fasting glucose, glycated hemoglobin, triglyceride, and serum creatinine (all *p* <0.001) while the lowest eGFR (*p* <0.001) (Table 1). Participants regardless of their groups were non-frail at baseline, with low prevalence of each FRAIL component (all <20%).

**Table 1:** Clinical features of participants with diabetes mellitus.

	No GLD (n = 21,160)	oGLD monotherapy (n = 11,901)	oGLD combination (n = 14,097)	Insulin users (n = 2,361)	*p-value*
*Demographic and physical data*					
Age (years)	64.0 ± 11.2	65.0 ± 11.2	64.0 ± 10.8	65.1 ± 10.9	<*0.001*
Sex (Female %)	10,281 (48.6)	5,535 (46.5)	6,478 (46.0)	1,142 (48.4)	<*0.001*
BMI (kg/m^2^)	24.9 (22.6, 27.5)	25.0 (22.7, 27.8)	25.1 (22.8, 27.8)	25.1 (22.5, 27.9)	*0.24*
*Comorbidity*					
Hypertension (%)	12,375 (58.5)	7,996 (67.2)	9,001 (63.9)	1,523 (64.5)	<*0.001*
Hyperlipidemia (%)	9,184 (43.4)	5,829 (49.0)	6,616 (46.9)	927 (39.3)	<*0.001*
Atrial fibrillation (%)	1,663 (7.9)	1,058 (8.9)	938 (6.7)	161 (6.8)	<*0.001*
Cerebrovascular accident (%)	846 (4.0)	646 (5.4)	669 (4.7)	105 (4.4)	<*0.001*
Cancer (%)	2,523 (11.9)	1,317 (11.1)	1,448 (10.3)	299 (12.7)	<*0.001*
Prior myocardial infarction (%)	374 (1.8)	361 (3.0)	450 (3.2)	91 (3.9)	<*0.001*
Acute coronary syndrome (%)	4,747 (22.4)	3,064 (25.7)	3,408 (24.2)	604 (25.6)	<*0.001*
Heart failure (%)	973 (4.6)	738 (6.2)	759 (5.4)	251 (10.6)	<*0.001*
Peripheral vascular disease (%)	514 (2.4)	288 (2.4)	285 (2.0)	89 (3.8)	<*0.001*
Chronic kidney disease (%)	2,563 (12.1)	1,549 (13.0)	1,596 (11.3)	690 (29.2)	<*0.001*
Chronic liver disease (%)	2,952 (14.0)	1,403 (11.8)	1,210 (8.6)	218 (9.2)	<*0.001*
COPD (%)	426 (2.0)	198 (1.7)	180 (1.3)	36 (1.5)	<*0.001*
Mental disorder (%)	357 (1.7)	186 (1.6)	160 (1.1)	32 (1.4)	<*0.001*
Malnutrition (%)	65 (0.3)	37 (0.3)	45 (0.3)	10 (0.4)	*0.82*
*Medication profile*					
Anti-hypertensives (%)	5,896 (27.9)	6,418 (53.9)	8,414 (59.7)	1,470 (62.3)	<*0.001*
Lipid-lowering agents (%)	2,711 (12.8)	3,313 (27.8)	4,728 (33.5)	799 (33.8)	<*0.001*
Anticoagulants (%)	367 (1.7)	350 (2.9)	313 (2.2)	57 (2.4)	<*0.001*
Anti-platelet agents (%)	3,326 (15.7)	3,492 (29.3)	4,646 (33.0)	776 (32.9)	<*0.001*
*Laboratory panel*					
Fasting glucose (mg/dL)	127.1 ± 45.3	127.6 ± 35.9	140.9 ± 47.5	152.2 ± 75.9	<*0.001*
Glycated hemoglobin (%)	6.89 ± 1.43	6.88 ± 1.21	7.35 ± 1.37	8.06 ± 1.76	<*0.001*
Total cholesterol (mg/dL)	186.0 ± 40.5	180.9 ± 40.2	180.1 ± 41.2	180.7 ± 47.9	<*0.001*
Triglyceride (mg/dL)	152.7 ± 111.2	157.7 ± 123.2	158.7 ± 108.3	163.2 ± 144.3	<*0.001*
Serum creatinine (mg/dL)	1.2 ± 1.2	1.2 ± 1.1	1.1 ± 0.8	1.8 ± 1.9	<*0.001*
eGFR (mL/min/1.73m^2^)	75.4 ± 24.9	73.9 ± 26.0	73.9 ± 25.2	60.0 ± 31.7	<*0.001*
*FRAIL component distribution*					
Fatigue	304 (1.4)	125 (1.1)	105 (0.7)	20 (0.8)	<*0.001*
Resistance	10 (0.04)	8 (0.07)	12 (0.09)	3 (0.1)	*0.36*
Ambulation	47 (0.2)	34 (0.3)	30 (0.2)	11 (0.5)	*0.09*
Illness	2,362 (11.2)	1,465 (12.3)	1,305 (9.3)	457 (19.4)	<*0.001*
Loss of body weight	597 (2.8)	234 (2.0)	242 (1.7)	31 (1.3)	<*0.001*
*Median duration of follow-up (with IQR) (years)*	*4.10 (2.0, 7.1)*	*4.02 (1.8, 7.3)*	*4.07 (1.7, 7.6)*	*2.94 (1.3, 6.0)*	

BMI, body mass index; COPD, chronic obstructive pulmonary disease; eGFR, estimated glomerular filtration rate; GLD, glucose-lowering drug; IQR, interquartile range; oGLD, oral glucose lowering drug

**Figure 2.**
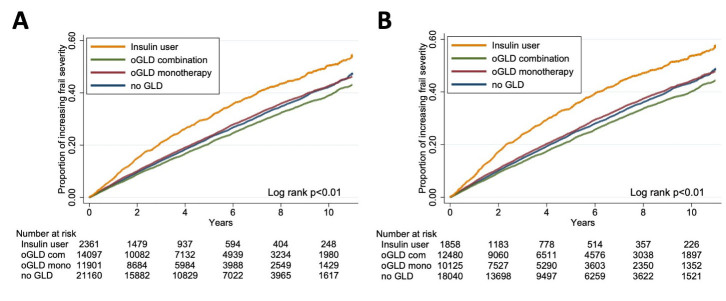
Kaplan-Meier event curves for increasing frail severity for groups adopting different glucose lowering strategies. (A) all participants; (B) subgroup without any positive FRAIL component. *GLD, glucose lowering drug; oGLD, oral glucose lowering drug.*

After 4 years of follow-up, 12,295 (24.8%) patients with DM had increasing frail severity over time (Table 2), among whom 24.7%, 26.0%, 22.0%, and 29.5% of the no GLD, oGLD monotherapy, oGLD combination, and the insulin user group participants developed the outcome of interest, respectively (Fig. 2A). Univariate analyses showed that oGLD combination group had significantly lower risk of having increased frail severity (HR 0.91, 95% CI 0.87 - 0.95), while insulin user group had significantly higher risk (HR 1.38, 95% 1.27-1.49) (Table 2). After adjusting for demographic profile, fasting glucose, and glycated hemoglobin, oGLD combination group remained at a lower risk (HR 0.87, 95% CI 0.83 - 0.91), while the risk of insulin user group persisted as well (HR 1.23, 95% 1.14 - 1.34). We further adjusted for other variables listed in Table 1 except mental disorder and malnutrition, and the results were not altered (Table 2). Finally, we examined the results after adjusting for mental disorder and malnutrition, with similar findings obtained (Table 2). Interestingly, when we divided oGLD combination group participants into subgroups receiving 2 oGLD and those receiving >2 oGLD, these two subgroups remained to have a significantly lower risk of rising frailty severity over follow up, with a trend toward less risk reduction in those receiving > 2 oGLD (Table 2).

**Table 2:** Risk of increasing frail severity according to the anti-diabetic regimen.

Variables	Number of events	Total population	Person-year	Incidence density^*^	Crude		Model A^#^		Model B^$^		Model C^&^	

					HR	95% CI	HR	95% CI	HR	95% CI	HR	95% CI
*Increasing frail severity*												
Without oGLD	5,224	21,160	98,693.1	52.93	1.00		1.00		1.00		1.00	
With oGLD monotherapy	3,089	11,901	56,354.3	54.81	1.03	0.99 - 1.08	0.98	0.94 - 1.03	0.99	0.95 - 1.04	0.99	0.95 - 1.04
With oGLD combination	3,285	14,097	67,854.3	48.41	0.91	0.87 - 0.95^†^	0.87	0.83 - 0.91^†^	0.90	0.86 - 0.94^†^	0.90	0.86 - 0.94^†^
*2 oGLD*	2,283	9,380	46,835.8	48.75	0.91	0.87 - 0.96^†^	0.87	0.83 - 0.92^†^	0.89	0.85 - 0.94^†^	0.89	0.85 - 0.94^†^
>*2 oGLD*	1,002	4,717	21,018.5	47.67	0.89	0.84 - 0.96^††^	0.87	0.81 - 0.93^†^	0.92	0.86 - 0.99^†††^	0.92	0.86 - 0.99^†††^
Insulin users	697	2,361	9,523.2	73.19	1.38	1.27 - 1.49^†^	1.23	1.14 - 1.34^†^	1.11	1.02 - 1.21^†††^	1.11	1.02 - 1.21^†††^

*CI, confidence interval; HR, hazard ratio; oGLD, oral glucose lowering drug*

* per 1000 patient-year

# Incorporating age, gender, glycated hemoglobin level, and fasting glucose

$ Incorporating model A variables and all others listed in Table 1 (except body mass index, mental disorder, and malnutrition)

& Incorporating model B variables and mental disorder, and malnutrition

† *p < 0.001*

†† *p < 0.01*

††† *p < 0.05*

Sensitivity analysis consisted of three parts: the first one focused on those without any FRAIL component at baseline. The Kaplan-Meier event curves of the 4 groups are illustrated in Figure 2B. We found that oGLD combination group still had a lower risk of having increased frail severity (HR 0.88, 95% CI 0.84 - 0.93), while the risk of insulin user group was still significantly increased (HR 1.16, 95% 1.06 - 1.26) (Table 3). Another sensitivity analysis addressed the influences of oGLD type, using metformin user as the reference. The Kaplan-Meier event curves of participants receiving different oGLD types are illustrated in Supplementary Figure S1. We showed that only dipeptidyl peptidase 4 inhibitor (DPP4i) users had a significantly higher risk of aggravating frailty over follow-up (HR 1.26, 95% CI 1.01 - 1.57), while other oGLD users did not (Supplementary Table 1). The third part of analysis focused on the influence of eGFR on study outcomes. We found that participants with declining eGFR levels had significantly higher risk of rising frail severity over time (Supplementary Table 2).

**Table 3:** Sensitivity analyses using participants without any FRAIL item at baseline.

Variables	Number of events	Total population	Person-year	Incidence density^*^	Crude		Model A^#^		Model B^$^		Model C^&^	
					HR	95% CI	HR	95% CI	HR	95% CI	HR	95% CI
*Increasing frailty severity*												
Without oGLD	4,810	18,040	86,275.2	55.75	1.00		1.00		1.00		1.00	
With oGLD monotherapy	2,889	10,125	49,688.9	58.14	1.04	0.99 - 1.09	0.99	0.94 - 1.03	0.97	0.93 - 1.02	0.97	0.93 - 1.02
With oGLD combination	3,135	12,480	61,700.3	50.75	0.90	0.87 - 0.95^†^	0.86	0.82 - 0.90^†^	0.88	0.84 - 0.93^†^	0.88	0.84 - 0.93^†^
Insulin users	644	1,858	7,856.9	81.97	1.46	1.35 - 1.59^†^	1.32	1.22 - 1.44^†^	1.16	1.06 - 1.26^††^	1.16	1.06 - 1.26^††^

*CI, confidence interval; HR, hazard ratio; oGLD, oral glucose lowering drug*

* per 1000 patient-year

# Incorporating age, gender, glycated hemoglobin level, and fasting glucose

$ Incorporating model A variables and all others listed in Table 1 (except body mass index, mental disorder, and malnutrition)

& Incorporating model B variables and mental disorder, and malnutrition

† *p < 0.001*

†† *p < 0.01*

## DISCUSSION

In this study, we followed up a large cohort of patients with type 2 DM for 4 years, analyzing their risk of having frail severity progression over time. After accounting for demographic features, comorbidities, medications, glycemic control levels and other laboratory variables, diabetic patients receiving oGLD combination had a significantly lower risk of developing frail severity progression compared to those without receiving any oGLD or received oGLD monotherapy. On the other hand, insulin users had an increased risk. In addition, the type of oGLD might play a role in modifying frailty progression risk. These findings stood regardless of baseline frailty status, glycemic control, and comorbidity severity. Judging from our findings, it would be important to consider the heterogenous influences on frailty risk imposed by GLD regimen in these patients, when it comes to medication reconciliation especially among those with DM and advanced age.

It is interesting to note that imbalances in comorbidities, medications, and laboratory profiles between groups receiving different glucose-lowering drugs existed. In Taiwan, insulin-naïve diabetic patients are frequently hesitant about insulin initiation and have poor compliance to insulin even if prescribed [25]. Insulin users may also have a protracted period of poor glycemic control before they start insulin treatment, as have been shown in a Pacific regional survey of patients with diabetes [26]. This phenomenon may be associated with findings that insulin users in Taiwan have significantly higher risk of developing diabetic complications such as retinopathy [27]. From this perspective, it can be expectable that in our cohort, insulin users could have significantly higher prevalence of most morbidities and organ complication, including diabetic nephropathy with resultant CKD.

Several prior studies used FRAIL scale to assess frailty trajectories among older adults. For example, Liu *et al*. in a community-based sampling of older adults, used FRAIL scale to evaluate changing frail severity over 2-year of follow-up [28]. They were able to show that one-third of participants had worsening frailty based on changing FRAIL item counts, while less than 10% had frailty regression. Another population-based study further used FRAIL scale scores as surrogates of frailty severity changes during decades of follow-up, exhibiting excellent associations with their functional status and morbidities [29]. These findings support the clinical applicability of FRAIL scale as a frailty trajectory surrogate. There are also studies harnessing other frailty-assessing instruments for gauging frailty trajectory, including modified Fried scale [30], frailty index [31], and several others. We will try other instruments in our future work and examine their applicability in our cohort.

Interestingly, we found that monotherapy of existing oGLD in patients with DM failed to reduce their risk of increasing frailty severity, while combinations of oGLD did (Table 2). Existing studies mostly addressed the pros and cons of individual GLD type in patients with DM, and there are concerns that intensification of GLD through polytherapy may compound treatment-related side effects in frail adults [32]. Indeed, metformin and α-glucosidase inhibitors can introduce adverse gastrointestinal effects, and persistent gastrointestinal symptoms may compromise oral intake and nutritional adequacy, causing treatment dilemma in vulnerable individuals. Deprescribing GLD in consideration of patients’ expected survival is an important issue in frail older adults, as is recommended by the American Diabetes Association [33]. However, under-treatment of otherwise non-frail older adults with DM should also be avoided, as urged by some experts [34], since treatment benefits potentially outweigh risk in this population. From this perspective, it would be tempting to infer that oGLD combination may confer therapeutic advantages through attenuating under-treatment probability compared to oGLD monotherapy or non-users, if we carefully select the population. Our participants were non-frail at baseline, and it is clear that the oGLD combination group had significantly higher glycated hemoglobin than the oGLD monotherapy or no GLD groups (Table 1), making them a suitable population for glycemic control optimization. In addition, a domestic study previously showed that oGLD combination may further improve glucose variability in type 2 DM patients compared to monotherapy receivers [35], and higher glucose variability is recently reported to correlate with increasing frailty prevalence among those with DM [36]. Consequently, oGLD combination may exert under-recognized benefits in those who are robust and have worse glycemic control, independent of other confounders.

Current evidence has not pinpointed the association between insulin use and frailty progression risk. We propose several plausible reasons to explain this relationship. First, GLD types differ with regard to their probability of precipitating hypoglycemia, whose frequency has been shown to correlate with frailty incidence [7]. A multi-national study disclosed that insulin users exhibited higher risk of hypoglycemia than oGLD combination users [37], suggesting that insulin use could indirectly modulate their frailty risk. Second, insulin users might harbor features that necessitate insulin use, including a greater degree of insulin resistance than oGLD users. Insulin resistance, accompanied by other immunometabolic dysfunction, jointly contributes to sarcopenia susceptibility, compromise in stress resistance, and later frailty occurrence [38]. Our findings support that monitoring the risk of frail severity increasing can be considered in insulin users. However, making the conclusion that insulin-based strategy should be deferred or withheld based on our observed phenomenon can be premature. More studies are needed to confirm our findings.

We also observed that DPP4i increased the risk of rising frail severity compared to metformin (Supplementary Table 1). DPP4i has been credited for its beneficial effects of hypoglycemia compared to sulfonylurea in frail adults [39]. However, a prior clinical trial showed that DPP4i increased the risk of hospitalization for heart failure among older adults with DM [40], and heart failure may well increase the risk of frailty in this population through multiple mechanisms [11]. A population-based study compared the cardiovascular risk between metformin and DPP4i users with DM, finding that DPP4i use was associated with a higher risk of cardiovascular events than metformin [41]. We believe that such excess risk might underly the increasing risk of rising frail severity among these patients in the long run. Indeed, researchers have lamented on the meager evidence for long-term benefits from DPP4i use besides glucose-lowering effect [42], and our findings may provide preliminary data supporting the re-consideration of long-term GLD options among older frail adults with DM.

We noticed that the fatigue component of the FRAIL scale was more prevalent in those of the no GLD group than in the other 3 groups (Table 1). Patients of the no GLD group also had a significantly higher prevalence of chronic liver disease and chronic obstructive pulmonary disease than the other 3 groups (Table 1). Prior literature revealed that patients with chronic liver disease, including cirrhosis, viral and alcoholic hepatitis, were at risk of having fatigue because of the associated pro-inflammatory status, mood disturbance, and impaired tissue metabolism [43]. Patients with chronic obstructive pulmonary disease are also shown to have higher prevalence of fatigue and higher fatigue severity than those without [44]. From this viewpoint, we believe that the reason for the higher prevalence of fatigue in those of the no GLD group may result from their increased prevalence of chronic liver disease and chronic obstructive pulmonary disease.

Our study has its strengths and limitations. We focused on an important yet rarely addressed issue during the pharmacological management of patients with DM, the risk of increasing frail severity. We showed that combinatorial oGLD could lessen the associated risk compared to no or monotherapy of oGLD, while inter-class risk differences could also be observed. These findings have not been reported before in the literature. In addition, our case number was large with a wide range of confounder accounted for, rendering our result of adequate validity. However, limitations also exist. First, we did not assess the risk of increasing frail severity among those with DM and pre-existing frailty, since their case number was too low to permit adequate statistical power for analysis. Second, our participants were middle-to-older age adults (>40 years) with type 2 DM. Whether our findings are applicable to adolescents and younger adults with DM, who are expected to sustain a longer period at risk of frailty development, is unknown. We did not have information regarding exercise habit or proteinuria status in these participants. Finally, we did not have data about the dosage of GLD in this study, and therefore we could not estimate whether there was a dose-response relationship. We are currently in the process of using alternative frailty assessment methods for verifying our findings.

## Conclusion

Using a large and representative cohort of patients with DM, we reported that different GLD strategies and even the type of oGLD might introduce variable influences on the risk of increasing frailty. oGLD combination exhibited a protective effect against rising frail risk, while insulin might work otherwise. DPP4i might significantly albeit mildly elevate the risk of increasing frailty compared to metformin. Based on our findings, oGLD combination may be considered for those with type 2 DM who are in a robust state to avoid their future risk of increasing frail severity. Individualized GLD adjustment is imperative if we wish to optimize patients’ functional outcome.

## Supplementary Materials

The Supplementary data can be found online at: www.aginganddisease.org/EN/10.14336/AD.2023.0225.



## Data Availability

The raw data for conducting this analysis are unavailable due to administrative regulations of NTUH-iMD.
