# Supplementary material for: The Impact of Glucose-Lowering Strategy on the Risk of Increasing Frailty Severity among 49,519 Patients with Diabetes Mellitus: A Longitudinal Cohort Study
Source: Aging Dis. 2023 Oct 1;14(5):1917–26. doi: 10.14336/AD.2023.0225 (PMC10529743; doi:10.14336/AD.2023.0225)
Supplement: Supplementary file 1 [file AD-14-5-1917-s.pdf]

SUPPLEMENTARY DATA

The Impact of Glucose-Lowering Strategy on the Risk of Increasing Frailty Severity among 49,519 Patients with Diabetes Mellitus: A Longitudinal Cohort Study

Chun-Yi Chi#, Jui Wang#, Szu-Ying Lee, Chia-Ter Chao*, Kuan-Yu Hung#, Kuo-Liong Chien

© 2023. Chi CY et al. Published online at http://www.aginganddisease.org/EN/10.14336/AD.2023.0225

SUPPLEMENTARY DATA

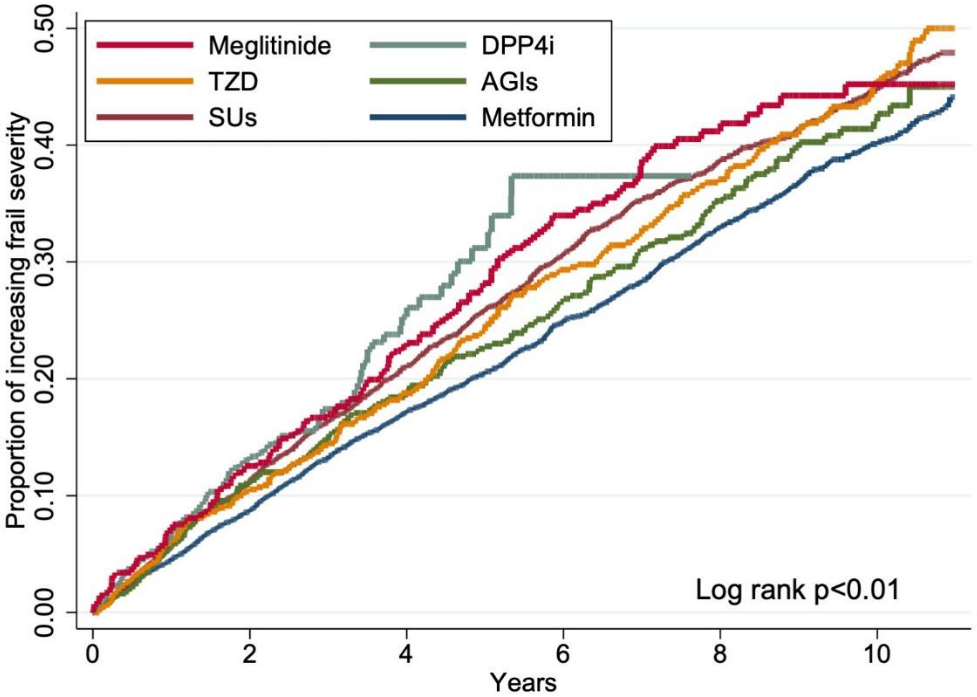

Supplementary Figure 1. Kaplan-Meier event curves for increasing frail severity for groups using different oGLDs. *AGI, alpha glucosidase inhibitor; DPP4i, dipeptidyl peptidase 4 inhibitor; oGLD, oral glucose lowering drug; SU, sulfonylurea; TZD, thiazolidinedione.*

Supplementary Table 1. Sensitivity analyses focusing on oGLD types

Variables	Number of events	Total population	Person-year	Incidence density*	Crude		Model A#		Model B$		Model C&	
					HR	95% CI	HR	95% CI	HR	95% CI	HR	95% CI
Increasing frailty severity												
Metformin	1,312	6,110	26,767.8	49.01	1.00		1.00		1.00		1.00	
Sulfonylurea	1,139	3,540	18,934.6	60.16	1.22	1.13 – 1.32†	1.09	1.01 – 1.18†††	0.99	0.91 – 1.08	1.00	0.92 – 1.08
α-glucosidase inhibitor	197	682	3,646.7	54.02	1.10	0.95 – 1.28	1.08	0.93 – 1.26	0.99	0.85 – 1.16	1.00	0.82 – 1.16
TZD	226	611	3,794.6	59.56	1.20	1.05 – 1.39††	1.17	1.01 – 1.35†††	1.07	0.93 – 1.23	1.07	0.92 – 1.23
DPP4i	89	537	1,242.7	71.62	1.49	1.20 – 1.85†	1.36	1.09 – 1.69††	1.26	1.01 – 1.57†††	1.26	1.01 – 1.57†††
Meglitinide	125	417	1,967.1	63.55	1.30	1.08 – 1.56††	1.19	0.99 – 1.43	1.04	0.87 – 1.26	1.04	0.87 – 1.26

CI, confidence interval; DPP4i, dipeptidyl peptidase 4 inhibitor; HR, hazard ratio; oGLD, oral glucose lowering drug; TZD, thiazolidinedione
* per 1000 patient-year
# Incorporating age, gender, glycated hemoglobin level, and fasting glucose
$ Incorporating model A variables and all others listed in Table 1 (except body mass index, mental disorder, and malnutrition)
& Incorporating model B variables and mental disorder, and malnutrition
† *$p < 0.001$*
†† *$p < 0.01$*
††† *$p < 0.05$*

© 2023. Chi CY et al. Published online at http://www.aginganddisease.org/EN/10.14336/AD.2023.0225

SUPPLEMENTARY DATA

Supplementary Table 2. Sensitivity analyses focusing on the influences of eGFR

Variables	Number of events	Total population	Person-year	Incidence density*	Crude		Model A#		Model B$		Model C&	
					HR	95% CI	HR	95% CI	HR	95% CI	HR	95% CI
Increasing frailty severity												
eGFR ≥ 60	7,401	36,643	178,161.9	41.54	1.00		1.00		1.00		1.00	
eGFR < 60 but ≥ 30	3,987	10,453	47,013.9	84.81	2.05	1.98 – 2.13†	1.57	1.51 – 1.63†	1.55	1.49 – 1.62†	1.55	1.49 – 1.62 †
eGFR < 30	907	2,423	7,249.1	125.12	3.10	2.90 – 3.33†	2.65	2.48 – 2.85†	2.43	2.18 – 2.72†	2.44	2.18 – 2.72 †

CI, confidence interval; eGFR, estimated glomerular filtration rate; HR, hazard ratio
* per 1000 patient-year
# Incorporating age, gender, glycated hemoglobin level, and fasting glucose
$ Incorporating model A variables and all others listed in Table 1 (except body mass index, mental disorder, and malnutrition)
& Incorporating model B variables and mental disorder, and malnutrition
† *p < 0.001*

© 2023. Chi CY et al. Published online at http://www.aginganddisease.org/EN/10.14336/AD.2023.0225
